# Insecticidal Activities of Bark, Leaf and Seed Extracts of *Zanthoxylum* *heitzii* against the African Malaria Vector *Anopheles gambiae*

**DOI:** 10.3390/molecules191221276

**Published:** 2014-12-17

**Authors:** Hans J. Overgaard, Patcharawan Sirisopa, Bertin Mikolo, Karl E. Malterud, Helle Wangensteen, Yuan-Feng Zou, Berit S. Paulsen, Daniel Massamba, Stephane Duchon, Vincent Corbel, Fabrice Chandre

**Affiliations:** 1 Department of Mathematical Sciences and Technology, Norwegian University of Life Sciences, P.O. Box 5003, Ås 1432, Norway; E-Mail: hans.overgaard@nmbu.no; 2 Department of Entomology, Faculty of Agriculture, Kasetsart University, 50 Ngam Wong Wan Rd, Ladyaow, Chatuchak, Bangkok 10900, Thailand; E-Mails: golf.patcharawan@gmail.com (P.S.); stephane.duchon@ird.fr (S.D.); 3 Institut de Recherche pour le Développement (IRD), Maladies Infectieuses et Vecteurs, Ecologie, Génétique, Evolution et Contrôle (IRD 224-CNRS 5290 UM1-UM2), Montpellier Cedex 5 34394, France; E-Mails: vincent.corbel@ird.fr (V.C.); Fabrice.Chandre@ird.fr (F.C.); 4 National Polytechnic High School, Marien Ngouabi University, BP 69, Brazzaville, Congo; E-Mails: mikolobertin@yahoo.fr (B.M.); danielmassamba@yahoo.fr (D.M.); 5 School of Pharmacy, Department of Pharmaceutical Chemistry, Section Pharmacognosy, University of Oslo, P.O. Box 1068 Blindern, Oslo 0316, Norway; E-Mails: helle.wangensteen@farmasi.uio.no (H.W.); yuanfengzou860315@163.com (Y.-F.Z.); b.s.paulsen@farmasi.uio.no (B.S.P.)

**Keywords:** *Zanthoxylum heitzii*, *Anopheles*, malaria, bark extract, insecticide

## Abstract

The olon tree, *Zanthoxylum heitzii* (syn. *Fagara heitzii*) is commonly found in the central-west African forests. In the Republic of Congo (Congo-Brazzaville) its bark is anecdotally reported to provide human protection against fleas. Here we assess the insecticidal activities of *Z. heitzii* stem bark, seed and leaf extracts against *Anopheles gambiae* s.s, the main malaria vector in Africa. Extracts were obtained by Accelerated Solvent Extraction (ASE) using solvents of different polarity and by classical Soxhlet extraction using hexane as solvent. The insecticidal effects of the crude extracts were evaluated using topical applications of insecticides on mosquitoes of a susceptible reference strain (Kisumu [Kis]), a strain homozygous for the L1014F kdr mutation (kdrKis), and a strain homozygous for the G119S Ace1R allele (AcerKis). The insecticidal activities were measured using LD_50_ and LD_95_ and active extracts were characterized by NMR spectroscopy and HPLC chromatography. Results show that the ASE hexane stem bark extract was the most effective compound against *An. gambiae* (LD_50_ = 102 ng/mg female), but was not as effective as common synthetic insecticides. Overall, there was no significant difference between the responses of the three mosquito strains to *Z. heitzii* extracts, indicating no cross resistance with conventional pesticides.

## 1. Introduction

Malaria is the single most important cause of ill health, death and poverty in sub-Saharan Africa [1,2]. Malaria vector control relies on insecticides for killing mosquitoes, thereby reducing man-mosquito contact and transmission [3]. Today, 14 insecticides from four major classes of synthetic chemical insecticides are recommended for indoor residual spraying (IRS) and six insecticides, all belonging to the pyrethroid group, are recommended for insecticide treated material (ITM) and long-lasting insecticide treated nets (LLIN) [4,5,6]. Constant exposure to insecticides invariably causes insecticide resistance [7]. A dramatic increase in pyrethroid resistance in malaria mosquitoes has been reported during the last decade and is now widespread throughout Africa [8]. Resistance is now considered a threat to current advances in malaria control [9,10]. One of the main challenges for vector control is, therefore, to preserve and improve current insecticide-based interventions, but also to develop a broader range of new insecticides with novel modes of action that can circumvent the current threat of insecticide resistance. Few new insecticides have been developed for public health in the last 30 years [9]. However, alternative candidates for vector control have been tested with promising results, such as dinotefuran [11], indoxacarb [12], and clorfenapyr [13]. All showed no cross resistance with the common resistance mutations, *kdr* and *ace1*, but had late-acting effect and kill insects at higher doses than conventional insecticides. As all recommended insecticides for vector control are nerve poisons being acetylcholinesterase (AChE) inhibitors or sodium channel modulators, excessive use may be harmful to humans and the environment, highlighting the need for safer and less expensive insecticides and repellents. Many current research initiatives aim at exploring natural plant extracts and their derivatives as sources of developing new anti-insect compounds. However, most such research has focused on larvicides and repellents, and relatively little work has been undertaken on adulticides [14].

The genus *Zanthoxylum*, syn. *Fagara*, belongs to the Rutaceae family and is found in tropical and subtropical regions around the world. It is a rich genus, with up to 250 identified species. Many of these species are traditionally used for controlling pests, infectious and metabolic diseases [15,16]. A considerable amount of research has been directed towards chemical analyses and biological activity of extracts and constituents of different plant parts of various species in the *Zanthoxylum* genus. Biological activity has been found against a wide range of organisms, including insects such as mosquitoes, beetles, cockroaches, and houseflies [17,18,19,20,21,22,23,24,25]. Alkaloids, amides, and terpenoids appear to be the substances most often implicated in anti-insect effects.

*Zanthoxylum heitzii* (Aubrév. & Pellegr.) P.G. Waterman, locally known as olon in the Republic of Congo, commonly occurs in the region from southern Cameroon to the Democratic Republic of Congo [26]. *Z. heitzii* has many traditional uses in Central and West Africa. Preparations from this plant have been reported to be used against a variety of diseases including jaundice [27], toothache [28], gonorrhea [29], and malaria [26]. It has also been reported to be used as fish poison [26].

Only a few investigations on chemical constituents and biological activity of *Z. heitzii* have been carried out, but alkaloids, lignans, triterpenes, esters and amides have been reported [30,31,32,33]. Hexane extracts from *Z. heitzii* bark was shown to be toxic to two weevil species and the American cockroach [34] and having antifilarial effect on the nematode *Loa loa* [35]. A preparation of *Z. heitzii* has been patented as an antiviral, antibacterial and immunostimulating remedy with possible anti-AIDS activity [36]. Methanol extracts (not further characterized) of bark of *Fagara heitzii* (a.k.a. *Z. heitzii*) induced >60% cell death in four out of five human cancer cell lines and also inhibited the growth of gram-positive bacteria [37].

However, no studies have been carried out so far on the adulticidal properties of *Z. heitzii* against malaria vector mosquitoes. Based on this background, we investigated the intrinsic insecticidal effect of *Z. heitzii* stem bark, seed and leaf extracts on adult *Anopheles gambiae* s.s. mosquitoes.

## 2. Results and Discussion

### 2.1. Extraction and Characterization of Active Fractions

The extract yields achieved by the two extraction methods and for the different plant parts are shown in Table 1. ^1^H- and ^13^C-NMR spectra of these extracts showed the presence of signals from aromatic, olefinic, oxygenated and aliphatic compounds. NMR spectra of Soxhlet and ASE hexane extracts were nearly identical (for NMR spectra, see Supplementary material, Figures S1–S4). From initial screening of extracts with HPLC-DAD (wavelength range of 200–400 nm), 237 nm was chosen as the reference wavelength as it gave the best profile of the constituents. The maximum wavelength at 237 nm could indicate that the major constituents of the bark hexane extracts are alkaloids (see below). The HPLC chromatograms at 237 nm showed some structural differences between the samples extracted by ASE and Soxhlet. The bark Soxhlet hexane extract gave a major peak (Rt 8.0 min) and three minor peaks (Rt 4.5, 6.7 and 7.6 min) (Supplementary material, Figure S5). The corresponding ASE hexane extract had the same major peak but the proportions between the constituents observed were different (Supplementary material, Figure S6). This could be explained by the different extraction yields of these two methods.

**Table 1:** Extraction yields (w/w) of *Zanthoxylum heitzii* stem bark, seeds and leaves by Soxhlet and Accelerated Solvent Extraction (ASE) methods. All extracts, except the Soxhlet hexane leaf extract, were used in the mosquito bioassays.

#	Extraction Method	Solvent	Bark	Seed	Leaf
1	Soxhlet	Hexane	2.0%	29.0%	2.7%
2	ASE	Hexane	0.75%	28.0%	2.3%
3	ASE	Ethyl acetate	0.4%	1.9%	1.5%
4	ASE	Ethanol (96%)	2.4%	7.9%	13.5%
5	ASE	Ethanol/water (50%/50%)	4.6%	11.1%	1.9%
6	ASE	Water (100%)	1.6%	2.3%	5.8%

### 2.2. Mosquito Bioassays

#### 2.2.1. General Toxicity

**Figure 1:**
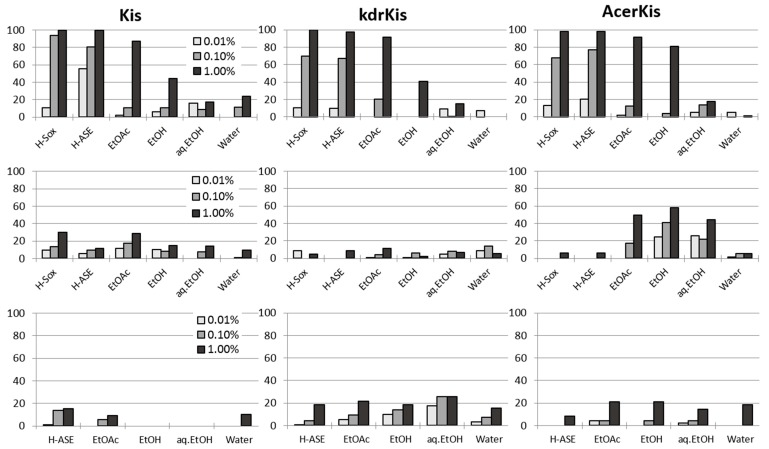
Mortality rates of three strains of *Anopheles gambiae* s.s. susceptible (Kis) (left panels), pyrethroid resistant (kdrKis; middle), and carbamate/organophosphate resistant (AcerKis; right) strains exposed by topical application to three concentrations (0.01%, 0.1%, and 1%) of crude extracts (extracted with hexane by Soxhlet (H-Sox) and with hexane (H-ASE), ethyl acetate, ethanol, ethanol/water and water by Accelerated Solvent Extraction) from stem bark (upper panels), seeds (middle panels), and leaves (lower panels) of *Z. heitzii.* The H-Sox extract of the leaves was not tested in this assay. Control mortalities: Mean = 4.0%, Median = 4.08%, Maximum = 6.2%, Minimum = 1.0%. All mortalities shown are corrected with Abbotts’ formula.

The stem bark extracts of *Z. heitzii* produced the highest mortalities in all mosquito strains and overall the hexane extracts were the most effective (Figure 1, Table 2). Mortality generally declined with increasing polarity of the solvent; water extracts producing the lowest mortalities. In the susceptible strain, 1% concentration of each bark hexane extract achieved 100% mortality, whereas 0.1% of the Soxhlet hexane extract killed >90% of mosquitoes and 0.1% of the ASE hexane extract killed about 80% of mosquitoes. In both resistant strains, the 0.1%-concentrations provided less than 80%, and 1%-bark hexane extract concentration was needed to achieve close to 100% mortality (Figure 1). Also the 1% bark ethyl acetate extracts produced >80% mortality in all mosquito strains. The toxicity of seed and leaf extracts was generally less than 30%, regardless of solvent, extraction method, or concentration (Figure 1). However, the AcerKis strain was slightly more sensitive compared to the other strains when exposed to 1% of seed extracts from ASE ethyl acetate, ethanol and ethanol/water solvents, producing mortalities ~50%.

#### 2.2.2. Toxicity to Hexane Bark Extracts

**Figure 2:**
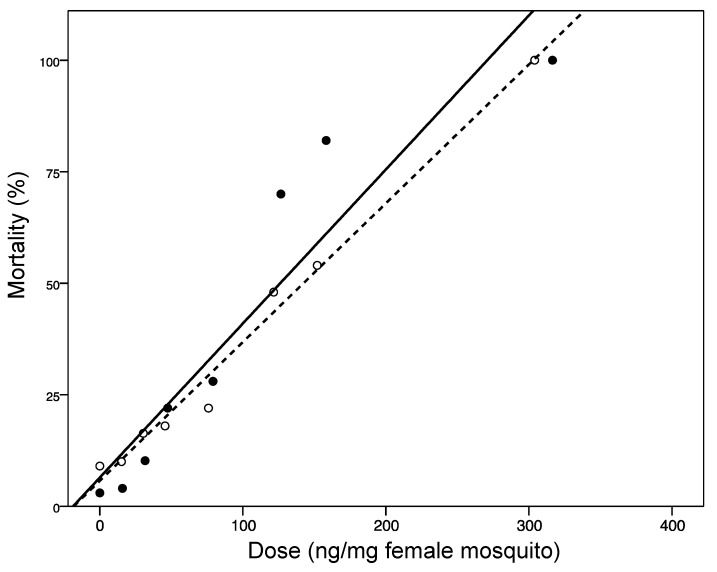
Probit transformed responses of *Z. heitzii* stem bark extracts on insecticide susceptible *An. gambiae* s.s. (Kis strain). Bark was extracted using Accelerated Solvent Extraction (ASE) with hexane solvent (filled circles, solid line: y = 6.43 + 0.35x; R^2^ = 0.88) and Soxhlet with hexane solvent (open circles, dashed line: y = 5.71 + 0.31x; R^2^ = 0.99).

The Pearson goodness-of-fit chi-square statistic showed an adequate fit of the factor data to the model (χ^2^ = 12.22, df = 10, *p* = 0.27). The parallelism test of the two factors was not significant (χ^2^ = 1.40, df = 1, *p* = 0.24), indicating that there is no significant difference between the slopes of the two lines, *i.e*., the relative responses of the two extracts are similar (Figure 2). However, the relative median potency of the ASE method is significantly higher than the Soxhlet method is (0.78, 95% CI 0.60–0.92) (confidence interval does not include 1). The dose-mortality relationships indicate that the ASE extract was slightly more toxic (LD_50_) than the Soxhlet extract against mosquitoes (Table 3).

**Table 2:** Mean mortality rates from general toxicity trial analyzed by ANOVA. The factor mortalities within each group reflect general mortalities which are influenced by other group factors. Therefore, mortalities do not indicate true mortalities, but are presented to show the relationships between each factor. For example, the real mortality of stem bark is much higher than 33.8% but is significantly different from seeds and leaves. The mosquito strains used were *Anopheles gambiae* s.s. susceptible (Kis), carbamate/organophosphate resistant (AcerKis), and pyrethroid resistant (kdrKis), and pyrethroid resistant (kdrKis).

Group	Factors	*n*	Mortality ^a^		df	F	*p*-Value
			%	95% C.I.			
Mosquito strain	Kis	51	18.4 ^§^	10.8–26.0	df_1_ = 2 df_2_ = 150	0.24	0.784
	AcerKis	51	19.9 ^§^	12.1–27.8			
	kdrKis	51	16.2 ^§^	9.3–23.2			
Plant part	Stem bark	54	33.8 ^†^	23.6–44.0	df_1_ = 2 df_2_ = 150	17.99	<0.0001
	Seed	54	11.1 ^‡^	7.6–14.7			
	Leaf	45	7.9 ^‡^	5.4–10.3			
Extract concentration	0.01%	51	6.4 ^α^	3.7–9.1	df_1_ = 2 df_2_ = 150	14.49	<0.0001
	0.10%	51	16.0 ^β^	9.3–22.7			
	1.00%	51	32.1 ^γ^	22.7–41.6			
Extraction method/solvent	Soxhlet/hexane	18	35.3 ^a^	15.5–55.2	df_1_ = 5 df_2_ = 147	3.91	0.002
	ASE/hexane	27	26.2 ^ac^	12.3–40.2			
	ASE/ethyl acetate	27	19.9 ^bc^	9.0–30.8			
	ASE/96% ethanol	27	15.5 ^bc^	7.4–23.6			
	ASE/50% ethanol-water	27	12.1 ^bd^	7.9–16.3			
	ASE/100% water	27	5.7 ^bd^	3.1–8.3			

^a^: Multiple comparisons by Duncan’s posthoc test. Within each factor, mortalities with different superscript symbols are significantly different from each other at the α = 0.05% level.

### 2.3. Discussion

We showed that crude extracts of *Zanthoxylum heitzii* plant parts kill *Anopheles gambiae* s.s., the primary vector of malaria in Sub-Saharan Africa. Insecticidal activity varied according to plant part, extraction method, solvent, and mosquito strain. Seed and leaf extracts produced very low mortalities, compared to the bark extracts, indicating that toxic compounds are mainly present in the bark. This is consistent with previous reports [34]. In our study, mortalities ranged from 97% to 100% in the three mosquito strains (insecticide susceptible and resistant) when exposed to the highest test concentration (1%) of Soxhlet and Accelerated Solvent Extraction (ASE) hexane stem bark extracts. The ASE bark extracts produced the highest mortality with an LD_50_ of 102 ng/mg female mosquito in the susceptible strain of *An. gambiae* s.s. In comparison, the Soxhlet extraction method gave an LD_50_ of 144 ng/mg female mosquito. We found no significant difference in mortality between the insecticide susceptible, the pyrethroid resistant, (L1014F mutation present), and the carbamate/organophosphate resistant (*Ace1^R^* mutation present) strains, indicating no cross resistance with conventional insecticides.

**Table 3:** Efficacy of *Z. heitzii* stem bark hexane extracts using Accelerated Solvent Extraction (ASE) and Soxhlet methods against susceptible (*kdr*) *An. gambiae* s.s. by topical application.

Extraction Method	Slope (±SD)	LD_50_ ng/mg Female (95% CI)	LD_95_ ng/mg Female (95% CI)
ASE	4.55 (1.07)	101.86 (74.75–117.93)	234.05 (191.85–379.84)
Soxhlet	6.50 (1.49)	144.45 (124.99–161.59)	258.71 (214.93–404.18)

Hexane proved to be the best solvent for extracting *Z. heitzii* compounds, thus showing that the active components are lipophilic. The NMR spectra of Soxhlet and ASE hexane extracts were almost identical, showing the presence of aromatic, olefinic, oxygenated and aliphatic compounds. The HPLC chromatograms indicated that the major constituents might be alkaloids. Alkaloids are well known in *Zanthoxylum* species [15].

The HPLC also showed some differences between the Soxhlet and ASE hexane extracts. While the components present appeared to be mostly the same, the ratios between them differed. This could potentially be explained by the different extraction yields (2% by Soxhlet and 0.75% by ASE). It is not clear from the chemical analyses why the mosquito bioassays showed a higher potency of the ASE extract than the Soxhlet extract. It could be that the higher extractive yield in the Soxhlet extraction gives more inactive material, thus diluting the active components. In this study, we only report results from crude extracts, but our further research will focus on identifying the most active fractions and compounds in the hexane extracts and testing them in mosquito bioassays.

Although *Z. heitzii* crude stem bark extract can kill adult female mosquitoes, it is not as effective as synthetic insecticides. As a comparison, studies have shown very low median lethal doses in *An. gambiae* exposed synthetic insecticides, e.g., 1.02 ng/mg female for permethrin, 0.018 ng/mg for deltamethrin, 0.14 ng/mg for bifenthrin, 0.04 ng/mg for fibronil, 0.34 ng/mg for dinetofuran, and 1.14–2.25 ng/mg for chlorpyrifos-methyl (depending on susceptible and *kdr* resistant strains) [11,38]. In another study, pyrethrum, also a natural product, had LD_50_’s of 1.9 ng/mg and 16.0 ng/mg in susceptible and pyrethroid resistant *An. gambiae*, respectively [39].

Apart from pyrethrum, it is not clear how well *Z. heitzii* performs as an adulticide compared to other botanical compounds. There is an emphasis in the botanical insecticide literature on larvicidal effects against malaria vectors, and reports on toxicity against adult mosquitoes are rare [14]. The focus on larvicides is potentially because of the comparative easiness of working with larvae. Larvicides have, however, so far not played a large role in malaria vector control. In an interim position statement, the WHO recommends use of larvicides in Sub-Saharan Africa in areas where breeding sites are few, fixed and findable [40]. In those investigations where adults have been tested, differences in methodology (e.g., topical application, adult bioassays) and reporting units (ng per mg mosquito, g per cm^2^ impregnated paper area, ppm) make comparisons difficult (e.g., [41]). To assess true (intrinsic) toxicity the topical application method should be used to avoid confusion with potential repellent effects. For example, in the WHO tube bioassays, mosquitoes may avoid treated paper surfaces by resting on the untreated areas on the tops and bottoms of tubes.

To our knowledge, this is the first study investigating the insecticidal activity of *Z. heitzii* against mosquitoes of public health importance. However, despite the urgent need to find new effective botanical and synthetic insecticides in the fight against malaria, the relatively low toxicity of *Z. heitzii* crude bark extracts to malaria mosquitoes could be an obstacle for further development. On the other hand, encouragingly, our findings point to no apparent cross resistance with conventional insecticides. In light of the rising problem of insecticide resistance, new insecticides are needed, and prospecting botanical resources should continue. Although crude plant extracts for insecticide control may have an important local role in control insect pests, the constituent compounds in potentially effective plants should be further investigated. Chemical synthesis of botanical insecticide analogues may also provide useful end products. Further research is required to elucidate details of the chemistry and biological activity of *Z. heitzii*. In addition to stem bark, seeds, and leaves, insect toxicity of other plant parts, such as flowers and root bark, should be investigated and tested on different mosquito species. Repellent and synergistic effects of constituent compounds should be analyzed and the mode of action of active compounds determined. Anecdotal reports say that *Z. heitzii* is toxic to fish [26]; therefore, the potential toxicity to non-target organisms as well as environmental persistence of *Z. heitzii* extracts need to be further investigated.

Finally, we agree with Isman and Grieneisen, [42] that much of the scientific literature on botanical insecticides is of limited use unless results can be reproduced and comparisons between studies made. A general research study protocol must be developed outlining detailed methodologies and standard operating procedures for botanical insecticide testing. The research community should agree on a common research agenda to advance botanical insecticide development and commercialization.

## 3. Experimental Section

### 3.1. Plant Material

Stem bark, seed and leaves of *Zanthoxylum heitzii* (Aubrév. & Pellegr.) P.G. Waterman (accepted name according to [43]) were collected near Douakani village in south-western Republic of Congo during the rainy season of 2011 (December). The plant material was identified using published keys [44] and voucher specimens were deposited at the National Herbarium, Centre of Study on Botanical Resources (CERVE), Brazzaville. Samples of the plant material are also deposited in the School of Pharmacy, Section of Pharmacognosy, University of Oslo, Norway (voucher numbers ZH-L111201, ZH-B111202, ZH-S111203).

### 3.2. Plant Preparation

Plant material was dried locally at room temperature for seven days and transported to the laboratory in the University of Oslo, Norway. Bark was cut into small pieces (<4 cm) and milled in a knife mill (Brabender, Duisburg, Germany) to pass through a 4-mm sieve. Seeds of *Z. heitzii* were milled in the same way as the bark.

### 3.3. Extraction

Extraction was conducted in May-June 2012 using two different methods, the classical Soxhlet method and the Accelerated Solvent Extraction (ASE) method, using an ASE350 Solvent Extractor (Dionex, Sunnyvale, CA, USA). Hexane was the only solvent used for the Soxhlet method, whereas five different solvents with increasing polarity (hexane, ethyl acetate, 96% ethanol, 50% water/ethanol and water) were used in the ASE method (Table 1).

Powdered plant material (250 g bark), was placed in a Soxhlet extractor and extracted with *n*-hexane (3.5 L) for 10 h. The extract was allowed to cool to room temperature and filtered using a Whatman No.1 filter paper. The extractor flask and filter were washed with dichloromethane and the washings combined with the filtrate. It was then taken to dryness on a rotary evaporator at 40–50 °C followed by 30 min on an oil vacuum pump.

In the ASE, powdered bark, seeds and leaves (100 g of each) were extracted successively with hexane, ethyl acetate, 96% ethanol, 50% water/ethanol and water (*ca.* 0.3 L of each). The solvents from the organic extractions were removed in vacuum on a rotary evaporator at 40 °C followed by 30 min in an oil pump vacuum. The extracts from 50% water/ethanol and water extractions were freeze-dried.

The Soxhlet and ASE hexane extracts (most active in bioassays) were characterized by nuclear magnetic resonance (NMR) spectroscopy and high performance liquid chromatography (HPLC). ^1^H- and ^13^C-NMR spectra were recorded at 300 and 75 MHz, respectively, in deuterochloroform on a Bruker DPX300 instrument (Bruker, Rheinstetten, Germany). HPLC analysis was performed on a LaChrom Elite HPLC system (VWR-Hitachi, Tokyo, Japan) equipped with an L-2455 diode array detector. A Chromolith Performance RP18e 100 × 4.6 mm column (Merck, Darmstadt, Germany) was used for separation. Elution was performed using a gradient of mobile phase A (water) and mobile phase B (acetonitrile) with the following time schedule: 20% B, 0–1 min; 20%–95% B, 1–15 min; 95% B, 15–16 min. The concentration of injected samples was 0.5 mg/mL, injection volume was 10 µL and flow rate was 3.0 mL/min. The absorbance was recorded at 237 nm, and separation took place at 25 °C. All samples were filtered (0.45 µm) prior to injection. The NMR spectra and HPLC chromatograms are appended as Supplementary material.

### 3.4. Mosquitoes

Three strains of *Anopheles gambiae* s.s. Giles were used in this study:

1. Kis: The Kisumu strain, originating from Kenya, is free of any detectable insecticide resistance mechanisms.
2. kdrKis: A pyrethroid and DDT resistant strain. This strain was obtained by introgression of L1014 F (*kdr*) into the genome of the susceptible Kisumu strain through successive backcrosses and selection with permethrin (1 mg/L). kdrKis has the same genetic background as the Kisumu strain but has the L1014F allele at the homozygous state.
3. AcerKis: An organophosphate and carbamate resistant strain. This strain was obtained by introgression of insensitive acetylcholinesterase (*Ace1^R^*) into the genome of the Kisumu strain through successive backcrosses and selection with propoxur (10 mg/L). AcerKis has the same genetic background as the Kisumu strain but differs by the presence of *Ace1^R^* allele (G119S) at homozygous state [45].

Mosquitoes were reared in a temperature-controlled space at 25 ± 5 °C, and 80% ± 10% relative humidity in the laboratory at the Institut de Recherche de Developpement (IRD), Montpellier, France. Strains were kept separately to ensure no accidental cross-breeding (contamination) between populations.

### 3.5. Mosquito Bioassays

Intrinsic insecticidal activity was assessed by topical application according to standard WHO protocol [3]. Two steps were carried out. In the first step, the general toxicity of bark, seed, and leaf extracts produced by the different extraction methods and solvents was evaluated against both susceptible and resistant mosquitoes. In the second step the toxicity of the most effective extracts was further evaluated to assess dose-effect relationships against susceptible mosquitoes. In each test twenty-five 2–5 days old non-blood fed female mosquitoes were anaesthetized with carbon dioxide and placed on a plate cooled at 4 °C.

Stock solutions were prepared at 1% by dissolving crude extracts in acetone (for hexane, ethyl acetate, and water extracts) and in ethanol (for ethanol and ethanol/water extracts). From the stock solutions, different concentrations were prepared and used in bioassays. A volume of 0.1 μL of stock solution was deposited on the upper part of the pronotum of each mosquito. Each test was duplicated giving a total of 50 mosquitoes per strain and per dose. After application of the solutions, females were transferred to plastic cups provided with honey-soaked cotton and maintained at controlled temperature (27 ± 2 °C) and humidity (80% ± 10%) conditions. Mortality rates were recorded after 24 h following tests and corrected using Abbott’s formula.

#### 3.5.1. Step 1—General Toxicity

First, an initial examination of the general level of toxicity was carried out. All three mosquito strains were exposed to three concentrations (0.01%, 0.1% and 1%) of each of the Soxhlet and ASE crude extracts of bark, seed and leaf of *Z. heitzii* (except Soxhlet hexane leaf extract). In addition, 2 × 50 females were treated with 0.1 μL of pure solvent to serve as controls.

#### 3.5.2. Step 2—Toxicity to Hexane Bark Extracts

Soxhlet and ASE hexane bark extracts, being most toxic (Figure 1), were selected for further examination of dose and mortality relationships. Only the susceptible *An. gambiae* s.s. Kis strain was used in this step. Female mosquitoes (2 × 25) were treated with 0.1 μL of each of seven concentrations (0.01%, 0.02%, 0.03%, 0.05%, 0.08%, 0.1% and 0.2%) of the two extracts. These concentrations produced a range of 0%–100% mortality. Four batches of 25 females each were used as controls.

### 3.6. Data Analysis

The comparison between each concentration of bark, seed and leaf extract from the initial examination (step 1) was analyzed by one-way ANOVA using SPSS version 15.0 (SPSS Inc., Chicago, IL, USA). Multiple comparisons between factor levels were done by Duncan’s posthoc test. In the second step the relationship between dose and mortality in the insecticide susceptible mosquito strain was analyzed by probit analysis using SPSS software (IBM SPSS Statistics for Windows, Version 21.0. IBM Corp., Armonk, NY, USA), which provides an estimation of the median lethal doses for each of the two compounds compared (Soxhlet and ASE bark *Z. heitzii* extracts). Median lethal doses (LD_50_ and LD_95_ with 95% confidence intervals) are expressed in nanograms of active ingredient per mg of average mosquito body weight (calculated from 50 live mosquitoes). The probit procedure further estimates the natural response rates, common slope and separate intercepts for each factor level. The Pearson goodness-of-fit chi-square statistic was used to test the null hypothesis that the model adequately fits the data. The relative median potency between the two compounds was compared and the parallelism was used to test if the assumption of equal slopes across factor levels is reasonable.

## 4. Conclusions

The olon tree, *Zanthoxylum* *heitzii*, is a traditional medicinal plant in Central and West Africa which also has insecticidal properties. Our research indicates that crude bark extracts of *Z. heitzii* is toxic to *Anopheles gambiae*, the main malaria vector in Africa. Although the insecticidal effect was lower than synthetic insecticides there was no apparent cross resistance with conventional insecticides. Further research is warranted to determine insecticidal efficacy and synergistic effects of the chemical constituents in the bark of *Z. heitzii*. We also encourage further studies on antiplasmodial activity of this species as it has been reported as being used as a malaria cure [26]. The toxicity to non-target organisms of various plant parts of this species also need to be investigated.

## Supplementary Materials

Supplementary materials (Figures S1–S6) can be accessed at: http://www.mdpi.com/1420-3049/19/12/21276/s1.
