# Supplementary material for: Insecticidal Activities of Bark, Leaf and Seed Extracts of Zanthoxylum heitzii against the African Malaria Vector Anopheles gambiae
Source: Molecules. 2014 Dec 17;19(12):21276–90. doi: 10.3390/molecules191221276 (PMC6270729; doi:10.3390/molecules191221276)

Supplementary Materials

Figure S1. ¹H-NMR spectrum of *Z. heitzii* bark extract—Soxhlet; hexane
Figure S2. ¹³C-NMR spectrum of Z. *heitzii* bark extract—Soxhlet; hexane
Figure S3. ¹H-NMR spectrum of Z. *heitzii* bark extract—ASE; hexane
Figure S4. ¹³C-NMR spectrum of Z. *heitzii* bark extract—ASE; hexane
Figure S5. HPLC chromatogram of Z. *heitzii* bark extract—Soxhlet; hexane
Figure S6. HPLC chromatogram of Z. *heitzii* bark extract—ASE; hexane
Please note: In ¹³C-NMR spectra, a noise artifact may often be observed at *ca.* 80 ppm.

Figure S1. ¹H-NMR spectrum of hexane extract (Soxhlet) of *Z. heitzii* bark.

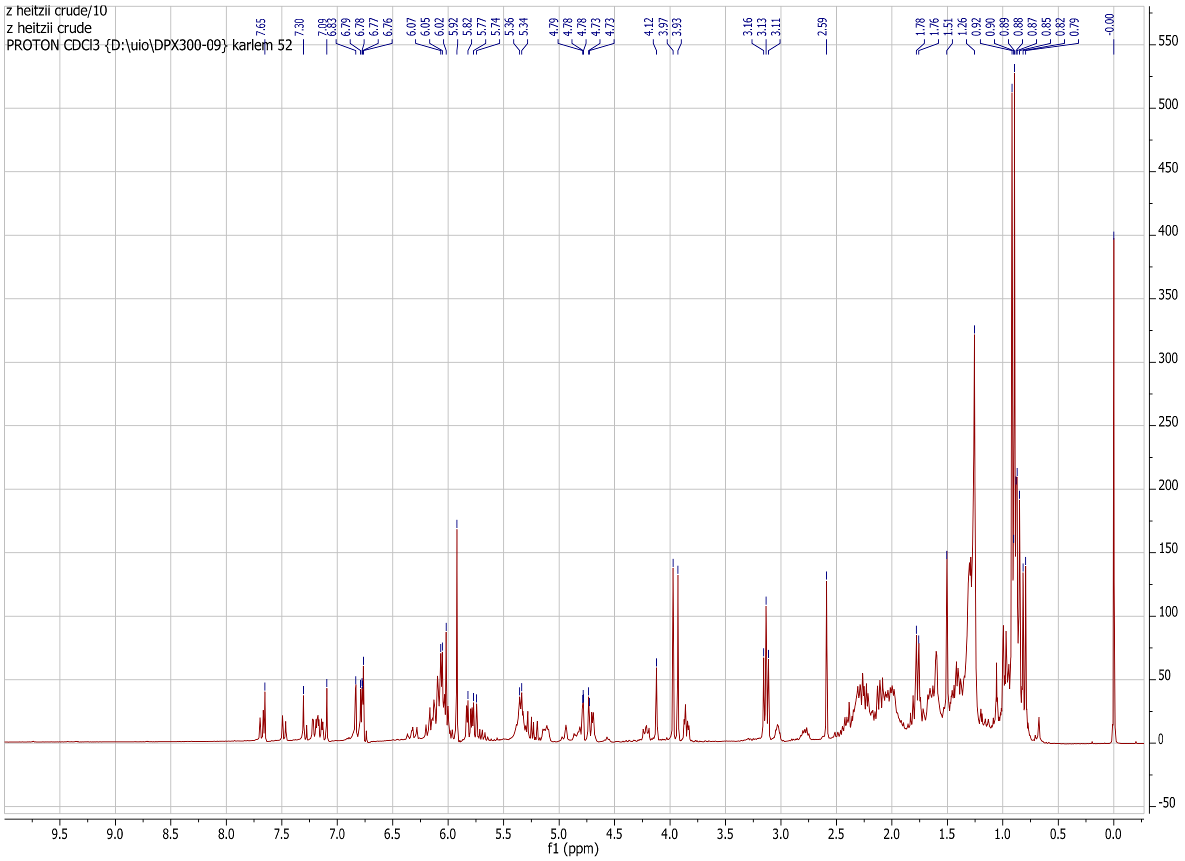

Figure S2. ^{13}C-NMR spectrum of hexane extract (Soxhlet) of *Z. heitzii* bark.

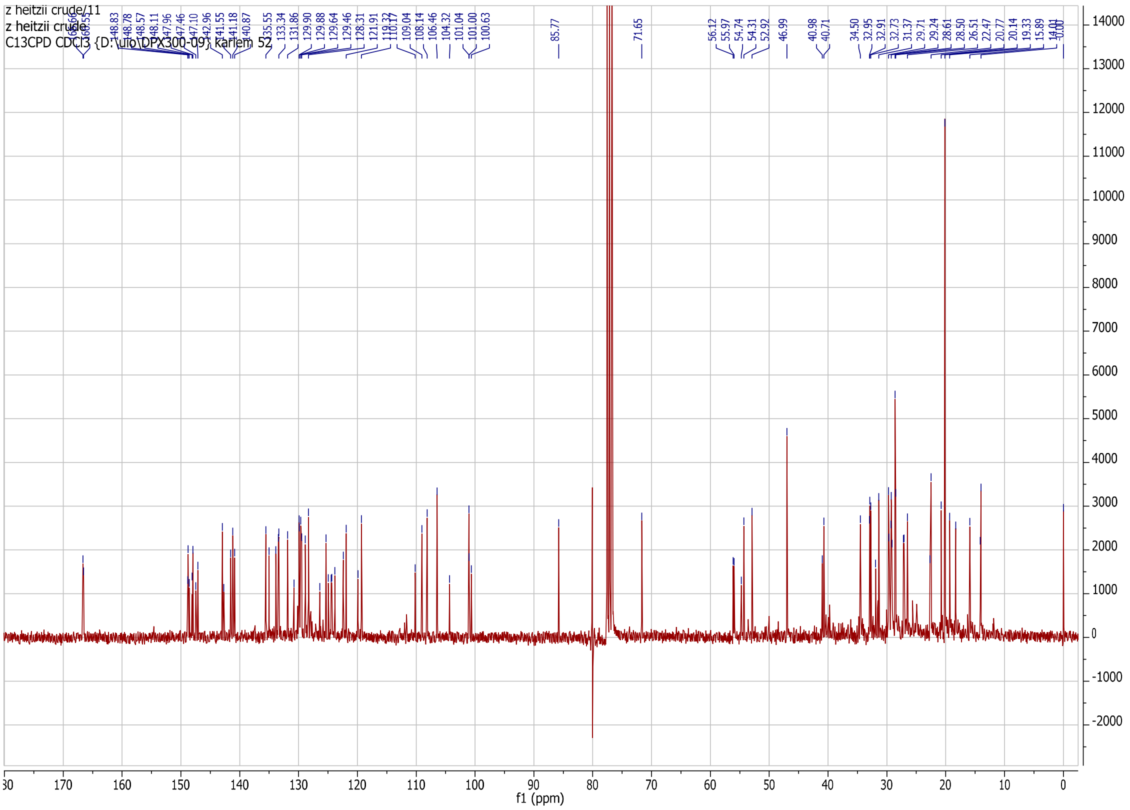

Figure S3. ^{1}H-NMR spectrum of hexane extract (ASE) of *Z. heitzii* bark.

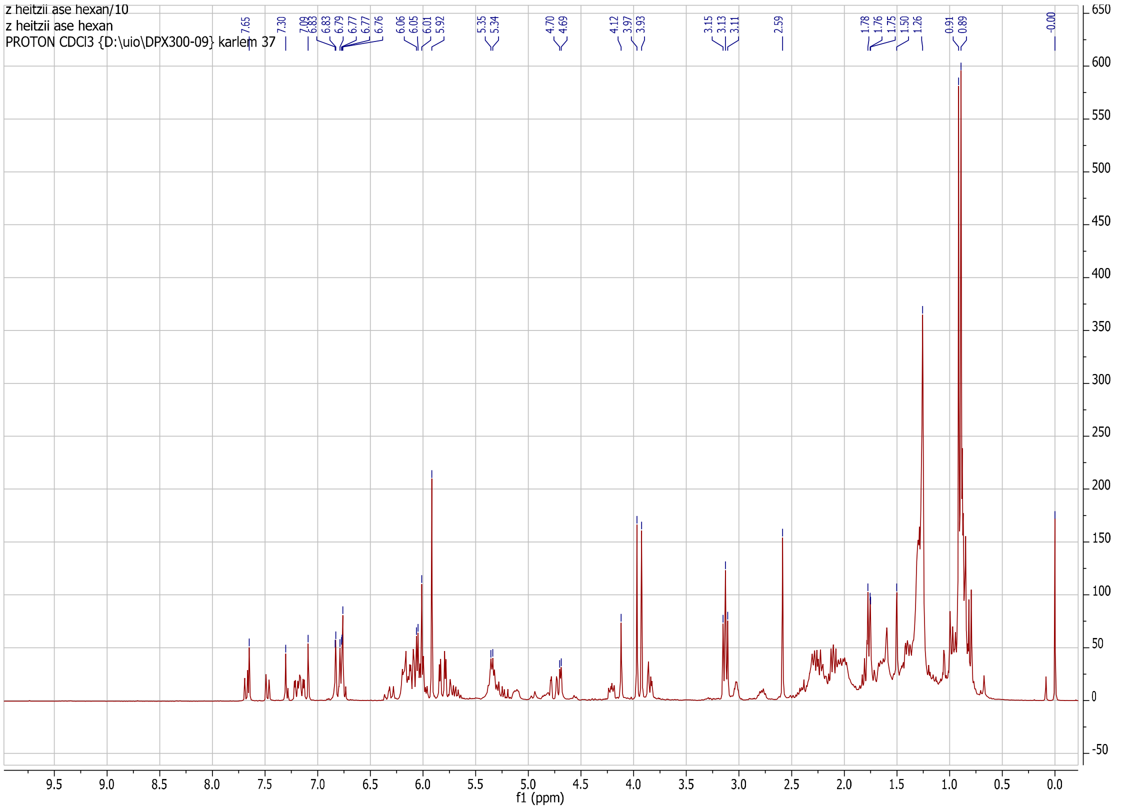

Figure S4. ^{13}C-NMR spectrum of hexane extract (ASE) of *Z. heitzii* bark.

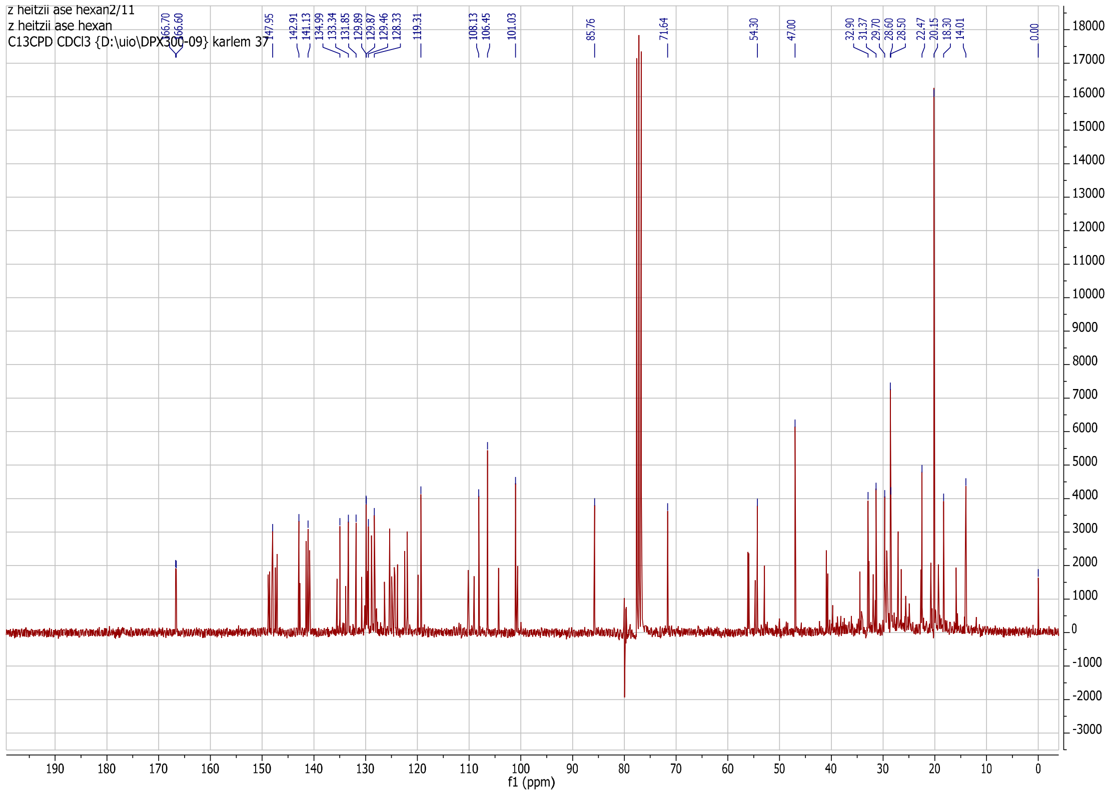

Figure S5. HPLC chromatogram of Z. *heitzii* bark extract—Soxhlet; hexane.

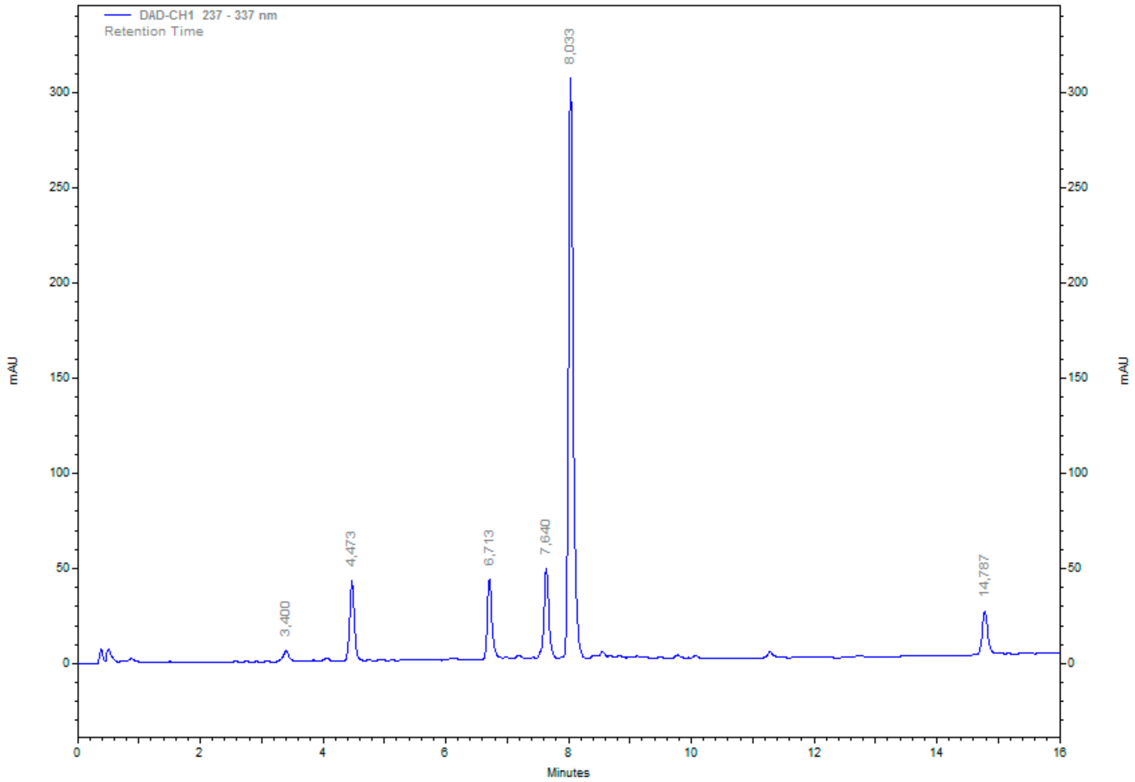

Figure S6. HPLC chromatogram of Z. *heitzii* bark extract—ASE; hexane.

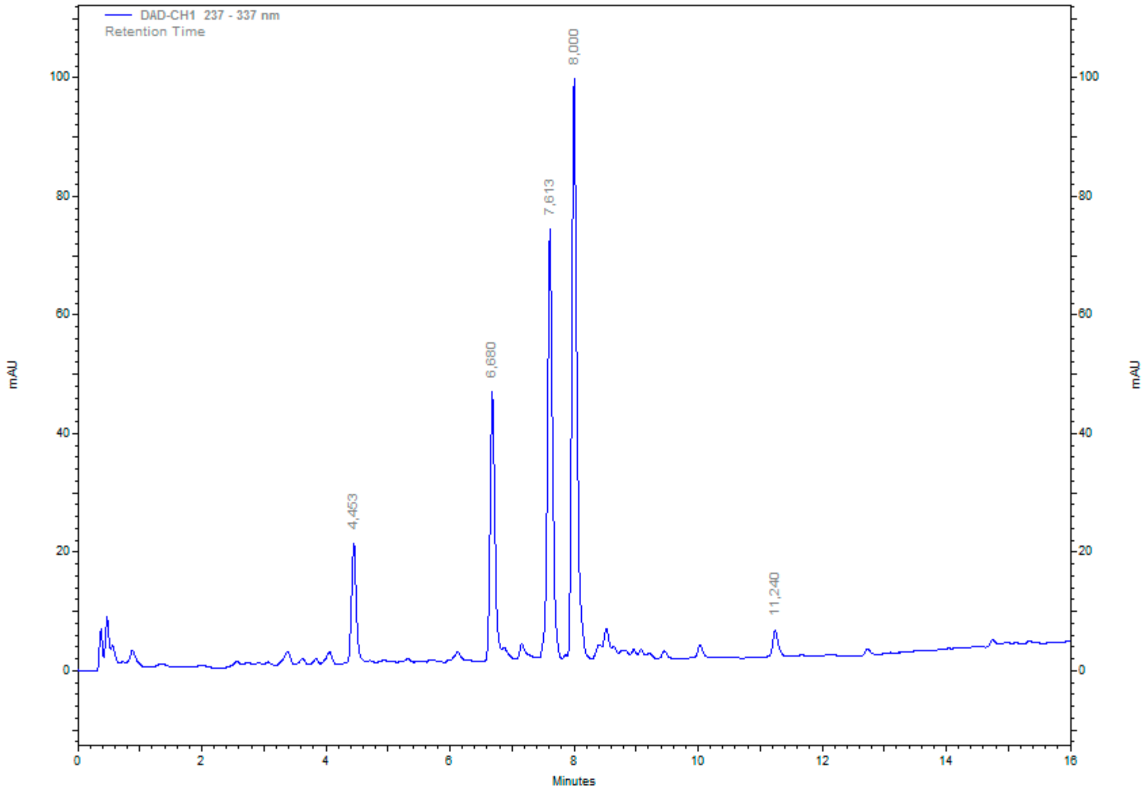

Supplement: Supplementary file 1 [file molecules-19-21276-s001.pdf]
